# Exendin-4, a glucagon-like peptide-1 analogue accelerates healing of chronic gastric ulcer in diabetic rats

**DOI:** 10.1371/journal.pone.0187434

**Published:** 2017-11-02

**Authors:** Yen-Cheng Chen, Ching-Chun Ho, Chih-Hsun Yi, Xiu-Zhu Liu, Tzu-Ting Cheng, Chen-Fuh Lam

**Affiliations:** 1 Department of Surgery, Buddhist Tzu Chi General Hospital, Hualien, Taiwan; 2 Department of Internal Medicine, Buddhist Tzu Chi General Hospital, Hualien, Taiwan; 3 Department of Anesthesiology, Buddhist Tzu-Chi General Hospital, Hualien, Taiwan; 4 Department of Anesthesiology, E-Da Hospital/E-Da Cancer Hospital/I-Shou University, Kaohsiung, Taiwan; Wayne State University, UNITED STATES

## Abstract

**Background:**

Diabetes mellitus is an independent risk factor for impaired healing of peptic ulcers, and there are currently no supplementary therapeutics other than the standard antipeptic medicine to improve the ulcer healing in diabetes. This study examined the potential pleiotropic effect of a glucagon-like peptide (Glp)-1 analogue exendin (Ex)-4 on the regeneration of gastric ulcer in streptozotocin-induced diabetic rats.

**Methods and results:**

Chronic ulcer was created in rat stomach by submucosal injection of acetic acid and peri-ulcer tissues were analyzed 7 days after operation. Ulcer wound healing was impaired in diabetic rats with suppressed tissue expression of eNOS and enhanced levels of pro-inflammatory reactions. Treatment with intraperitoneal injection of Ex4 (0.5 μg/kg/d) significantly reduced the area of gastric ulcer without changing blood glucose level. Ex-4 restored the expression of pro-angiogenic factors, and attenuated the generation of regional inflammation and superoxide anions. The improvement of ulcer healing was associated with increased expression of MMP-2 and formation of granulation tissue in the peri-ulcer area.

**Conclusion:**

Administration of Ex4 may induce pro-angiogenic, anti-inflammatory and anti-oxidative reactions in the peri-ulcer tissue of diabetic rats that eventually enhances tissue granulation and closure of ulcerative wounds. Our results support the potential clinical application of Glp-1 analogues as supplementary hypoglycemic agents in the antipeptic ulcer medication in diabetes.

## Introduction

Impaired wound healing in diabetic patients is a considerable burden in clinical care due to susceptible to infection, increased generation of advanced glycation products and delayed formation of granulation tissue. Persistence of inflammation and neutrophil infiltration is typically characterized by upregulation of pro-inflammatory mediators (such as IL-1, TNF-α, IL-6, RANTES and superoxide anions), and increased levels of nitrosative and oxidative stress generated by the activated monocytes and leukocytes in diabetic subjects [1]. Hyperglycemia has also known to impair vascular endothelial function and suppress pro-angiogenic effect [2]. Diabetes not only impairs the healing process of cutaneous wounds, prevalence of peptic ulcer disease (PUD) is also significantly higher in patients with diabetes (8.5 vs 14.8%, P< 0.001) [3]. In a large-scale population-based study, diabetes was found as an independent risk factor for peptic ulcer bleeding (hazard ratio 1.44, 95% confidence interval 1.11–1.86; P< 0.001) [4]. Healing of iatrogenic ulcer resulted from endoscopic dissection of mucosa was prolonged (>3 months) in the presence of diabetes mellitus [5]. Experimental models also demonstrated that peptic ulcer healing was significantly reduced by 300% in diabetic rats with decrease in the gastric mucosal blood flow [6]. Although these clinical and experimental investigations underscore that diabetes delays the healing process of peptic ulcers, there are currently no effective clinical therapeutic approaches in addition to the standard antipetic ulcer medicine to accelerate the recovery of PUD in diabetic patients.

Glucagon-like peptide-1 (Glp-1) is a gut-derived hormone belongs to the incretin family, which stimulates the release of insulin through the binding to Glp-1 receptor (Glp-1R) on pancreatic beta cells [6], and have been applied to the treatment of type II diabetes [7]. Glp-1 and its agonists have been reported to exert cardioprotective activity in recent preclinical and clinical studies, including coronary artery disease [8]. The cardiovascular protective effect of Glp-1 is attributed to suppression of endothelial cell apoptosis [9] and amelioration of oxidative stress-induced vascular endothelial dysfunction [10]. We have recently discovered that treatment with exendin-4 (Ex4, a Glp-1R analogue) enhanced the excisional wound closure in diabetic rats, and significantly suppressed the tissue generation of superoxide anions and interleukin (IL)-6 [11]. Exendin-4 mobilized circulating endothelial progenitor CD34^+^/KDR^+^ cells and enhanced expressions of proangiogenic factors in the cutaneous wounds [11]. Additionally, the pharmacological pleiotropic properties of Glp-1R analogues may also provide beneficial effects on gastrointestinal system [12,13]. Extended logically from these findings, we speculated that the potent anti-inflammatory and proangiogenic effects mediated by Glp-1R analogues might potentiate the healing of gastric ulcer in rats with experimental diabetes.

## Materials and methods

### Diabetic rat model of chronic peptic ulcer

All animal experimental procedures were approved by the Institutional Animal Care and Use Committee (IACUC; Tzu Chi Medical University, Hualien, Taiwan) and were performed in accordance with the Guide and Use of Laboratory Animals (Institute of Laboratory Animal Resources). Diabetes was induced in male Sprague-Dawley rats (weight approximately 250 g) by a single dose intraperitoneal injection of streptozotocin (65 mg/kg). Increased blood glucose levels (>250 mg/dl) on day 7 after streptozotocin injection confirmed the development of diabetes. Rats were anesthetized with inhaled desflurane (6–8% v/v in oxygen) and the anterior wall of stomach was exposed following midline abdominal incision. Acetic acid (0.05 ml, 100 v/v%) was injected into the submucosal layer of the antrum portion of the stomach using a 0.1-ml microsyringe, followed by saline irrigation to avoid formation of adherence [14]. The abdominal wall was then closed in layers and the animals were recovered from anesthesia in a heated cage. Animals were fed with the original chow 6 hours after operation.

### Treatment protocol

Rats were randomly assigned into four treatment groups at the beginning of study, as sham, control, control+Ex4, DM, and DM+Ex4 groups. Sham-operated animals received only laparotomy and exposure of stomach, while all other animals received operation for creation of gastric ulcer. Exendin-4 (0.5 μg/kg/d) was administered intraperitoneally in the Ex4 treated groups for up to POD7 [11]. Daily oral intake and body weight was recorded. Animals were euthanized at POD7, and blood samples and peri-ulcer gastric tissues were harvested.

### Measurement of gastric ulcer

Immediately after euthanasia, the stomach was dissected to expose the ulcer on the antrum. The pH of gastric juice was determined by a pH meter. The gastric ulcer was photographed and areas of ulcer (mucosal and basal margins) were quantified using the ImageJ Software (1.48v, National Institutes of Health, USA). Peri-ulcer tissues (surrounding width of 1 cm) were obtained for analysis.

### Biochemical analysis of blood and gastric tissue samples

The blood and tissue levels of inflammatory cytokines (IL-1β, IL-6, IL-10 and MCP-1) were determined using rat cytokine ELISA assay kits (Signosis Inc.). Concentrations of cAMP in the gastric tissues were measured by an ELISA kit (R&D System, USA). The release of superoxide anions from the gastric tissue samples was determined by a luminol-enhanced chemiluminescence chamber (Tohoku Electronic CLA 2100, Sendai, Japan) [15]. Blood glucose levels were analyzed by the colorimetric assays (ADVIA 1800 Chemistry System, Siemens). All analysis procedures were performed in accordance to the manufacturer’s instructions.

### Western blot

Soluble protein extracts (50 μg) were loaded into polyacrylamide gels (9–12%) and transferred onto the nitrocellulose membranes. Membranes were immunoblotted overnight with mouse monoclonal anti-eNOS (1:2000; BD Transduction Labs), anti-peNOS (1:2000; BD Transduction Labs), anti-MMP-2 (1:2000; Millipore), anti-CD11b (1:3000; BD Biosciences), anti-HO-1 (1:3000; Stressgen), anti-IL-10 (1:1000; Abcam) and anti-cleaved caspase-3 (1:3000; BD Biosciences) antibodies. After washing, the membranes were incubated with horseradish peroxidase-linked secondary antibodies for 1 h at room temperature. Chemiluminescence enhanced bands were visualized using an automated imaging system (UVP Biospectrum). Protein levels were quantified by scanning densitometry (ImageJ Software; 1.48v, National Institutes of Health, USA).

### Myeloperoxidase activity assay

The gastric tissue was homogenized in phosphate buffer solution and suspended in 50 mM hexadecyltrimethylammonium bromide. After thawed and frozen for four times, the resulting samples were mixed with O-dianisidine dihydrochloride and hydrogen peroxide and the solution was proceeded for detection of absorbance at 460 nm at regular intervals for 2 minutes.

### Histological examination

Gastric biopsies were immersed in 10% formaldehyde for 24 hours. Paraffin-embedded tissues were sectioned and stained with HE, and Masson's trichrome stains. For immunohistochemical analysis, the sectioned tissues were incubated with rabbit polyclonal anti-myeloperoxidase antibody (1:50; BD Transduction Laboratories) to visualize the expression of myeloperoxidase using the avidin-biotin method.

### Statistical analysis

When experiments were performed in parallel on tissues from different groups of animals and the normality assumptions were met, one-way analysis of variance (ANOVA) were employed; otherwise the Kruskal-Wallis test was used, as appropriate. Multiple comparisons were performed using the post hoc Tukey test. A P value less than 0.05 was considered to be statistically different in all tests. All the statistical analyses were performed using the SigmaStat Scientific Software (version 2.0; Jandel Corporation, San Rafael, CA).

## Results

### General outcomes

The overall perioperative mortality of rats with gastric ulcer was 2.2% (one rat in the control group died on postoperative day (POD) 4 due to bowel perforation. The general physical conditions were assessed by changes of body weight after induction of chronic ulcer. The mean body mass was marginally increased in the control animals, but body mass loss was recorded in diabetic rats with or without receiving Ex4 treatment (Table 1). Blood glucose levels were significantly elevated in streptozotocin-induced diabetes, but administration of Ex4 (0.5 μg/kg/d) did not affect the glucose levels in these animals (Table 1). Regardless to the presence of diabetes or treatment drug, the pH values were reduced in rats with gastric ulcer (Table 1). Serum markers of pro-inflammatory cytokines IL-6 and MCP-1 were not significantly different between the study groups, but elevated serum concentrations of MCP-1 were noted in rats with diabetes (Table 1).

**Table 1:** Physiological parameters and blood tests.

Parameters	Sham	Control	Control+Ex4	DM	DM+Ex4
Body mass gained (g)*	-	+2.1±23	+11.1±9.9	-27.5±11.5	-18.0±7.5
Gastric pH	4.1±0.5	3.3±1.2	3.3±0.5	3.0±0.9	2.9±0.5
Blood glucose (mg/dl)^#^	166.2±33.9	153.5±22.5	121.5±12.1	329.6±125.5^†^	381.0±104.0^†^
Serum IL-6 (pg/ml)	35.5±11.5	46.2±11.9	-	36.7±13.7	52.6±46
Serum MCP-1 (pg/ml)	70.3±30.7	90.0±59.4	-	138.5±101.8	115.8±82.3

DM: diabetes mellitus; Ex4: exendin-4; IL-6: interleukin-6; MCP-1: monocyte chemotactic protein-1;

*Body mass (BM) change = BM at induction of peptic ulcer—BM at sacrifice.

^#^Blood glucose levels were measured at the end of experiment (at sacrifice).

^†^P< 0.05 compared with control (statistical analysis was performed with sham group). Serum concentrations of IL-6 and MCP-1 were not measured in control+Ex4 group. Data was analyzed by one-way ANOVA. n = 8–9 in control, control+Ex4, DM and DM+Ex4 groups, n = 4 in sham.

### Exendin-4 accelerated healing of peptic ulcer in diabetic rats

**Fig 1:**
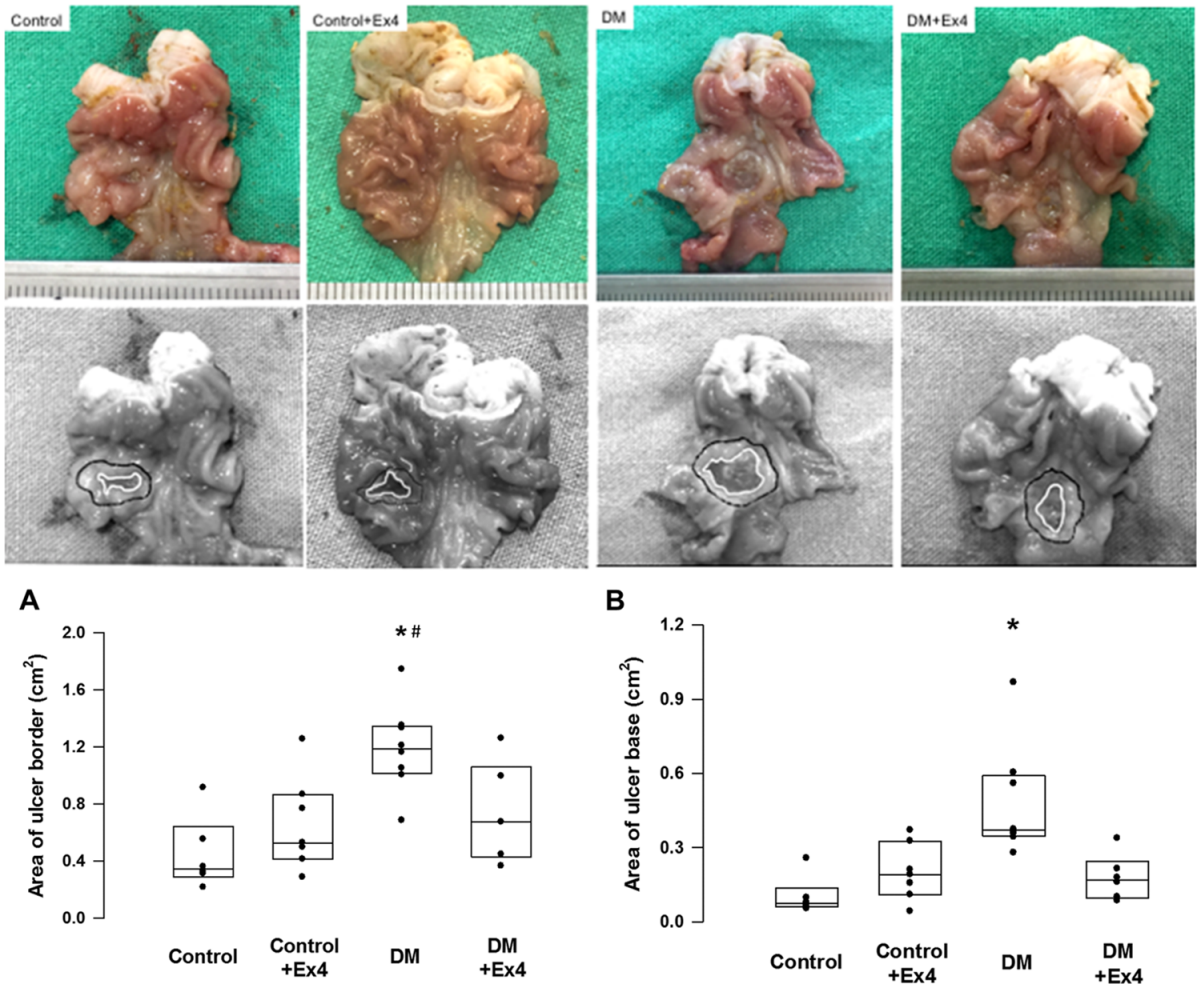
Representative photographies of gastric ulcer in controls and diabetic rats. Ulcer areas were determined by measuring the margins of mucosa (ulcer border) and ulcer base at day 7 after creation of gastric ulcer. Both ulcer border and ulcer base areas were significantly enlarged in diabetic rats, and were potentitated by exendin-4 (Ex4) treatment. The ulcer areas were quantified using the ImageJ Software. Data were analyzed by the one-way ANOVA, and are presented as median (interquartile range). *P< 0.05 DM vs control, ^#^P<0.05 DM vs DM+Ex4. n = 8–10 different animals in each group.

Submucosal injection of acetic acid induced chronic ulcer wounds in the gastric antrum with a mean wound border size of 0.45±0.26cm^2^ at POD 7 in non-diabetic controls (Fig 1). Diabetes significantly impaired the healing of ulcer wound, but treatment of Ex4 significantly accelerated the closure of ulcer margin (ulcer border size of 1.2±0.31cm^2^ vs 0.74±0.34cm^2^, respectively; P = 0.037) (Fig 1).

### Exendin-4 suppressed proinflammatory reaction in the chronic ulcer

**Fig 2:**
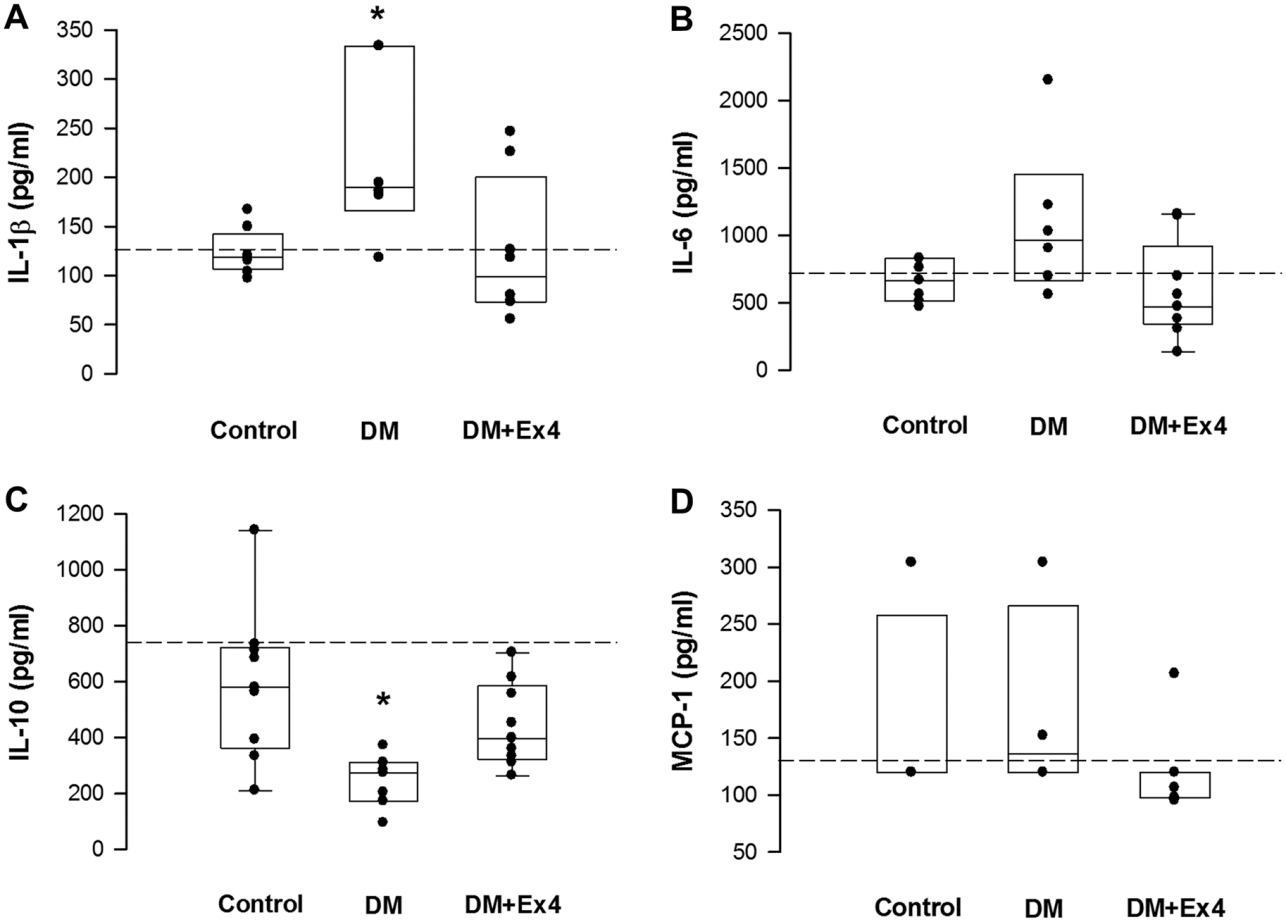
Quantification of tissue concentrations of inflammatory cytokines in the peri-ulcer gastric tissues at day 7 after creation of gastric ulcer in controls, diabetic (DM) rats and diabetic rats treated by exendin-4 (DM+Ex4) using ELISA kits. Concentrations of interleukin (IL)-1β, IL-6, IL-10 and monocyte chemotactic protein (MCP)-1 were analyzed. Data were analyzed by the one-way ANOVA, and are presented as median (interquartile range). *P< 0.05 DM vs DM+Ex4 in IL-1β, and DM vs control in IL-10. n = 4–9 different animals in each group. Dashed lines indicate the mean concentrations of sham-operated animals.

**Fig 3:**
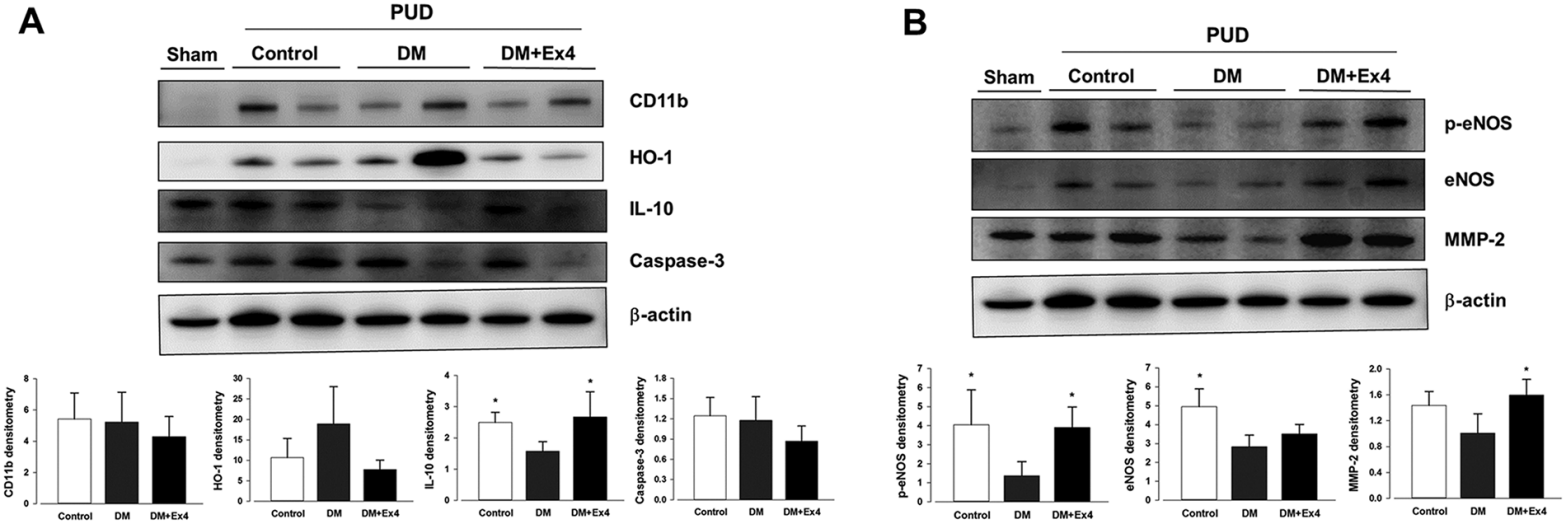
Expressions of inflammation-related (A) and angiogenesis/remodeling-related (B) proteins in the peri-ulcer gastric tissues obtained from the three treatment groups at day 7 after creation of gastric ulcer. Ex4: exendin-4, HO-1: heme oxygenase-1, IL-10: interleukin-10, eNOS: endothelial nitric oxide synthase, peNOS: phosphorylated eNOS, MMP-2: matrix melloproteinase-2. Western blots were quantified by scanning densitometry (ImageJ Software). Data were analyzed by the one-way ANOVA, and are presented as mean±SD. *P< 0.05 in two groups. n = 5–6 different animals in each group.

**Fig 4:**
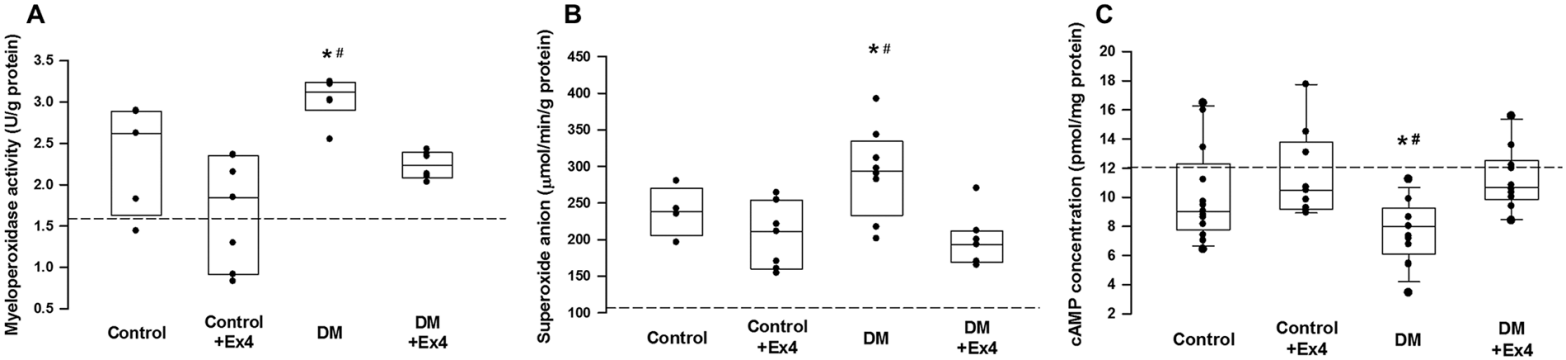
**4A.** Measurement of myeloperoxidase activity in the peri-ulcer tissues of the three treatment groups at day 7 after creation of gastric ulcer. n = 5–6 different animals in each group. **4B.** Measurement of tissue levels of superoxide anions in the peri-ulcer tissues. n = 5–8 different animals in each group. **4C.** Measurement of concentrations of cyclic adenosine monophosphatase (cAMP) in the peri-ulcer tissues. n = 9–13 different animals in each group. Data were analyzed by the one-way ANOVA, and are presented as median (interquartile range). *P< 0.05 DM vs DM+Ex4, ^#^P< 0.05 DM vs control+Ex4. Dashed lines indicate the mean concentrations of sham-operated animals.

The regional inflammatory reaction in the peri-ulcer gastric tissue was determined by the levels of pro-inflammatory cytokines, superoxide anions, and activity of myeloperoxidase. Tissue concentration of IL-1β was significantly enhanced in diabetic rats, but was suppressed to the similar levels of controls following treatment with Ex4 (Fig 2). Level of IL-6 in the gastric tissue of diabetic animals was also attenuated by Ex4, but the difference was not statistically significant (Fig 2). On the other hand, the tissue content of IL-10 was reduced in diabetes, but was restored in the Ex4 treated rats (Fig 2). Consistent with the quantitative assay, the protein expression of IL-10 was suppressed in diabetes and was enhanced in diabetic animals treated with Ex4 (Fig 3A). On the other hand, the expressions of CD11b (a cell marker for activated leukocytes), heme oxygenase-1 (an inducible anti-inflammatory protein) and caspase-3 (a cell marker for apoptosis [16]) were not significantly different between the three groups (Fig 3A). Nevertheless, the up-regulated activity of myeloperoxidase and tissue content of superoxide anions in the peri-ulcer tissue of diabetic rats was mitigated following Ex4 treatment (Fig 4A and 4B).

### Exendin-4 enhanced pro-angiogenic factors in the chronic ulcer

The protein expressions of endothelial nitric oxide synthase (eNOS) and its phosphorylated form (peNOS) in the ulcer tissue were suppressed in animals with diabetes, but they were significantly enhanced after treatment with Ex4 (Fig 3B). Furthermore, the expression of active matrix metalloproteinase (MMP)-2, an important spatial gelatinase during healing of PUD [17], was restored in diabetic animals received Ex4 treatment (Fig 3B). Compared with controls, the bioavailability of cAMP in the ulcer tissue was significantly reduced in diabetic rats and was potentiated to the control level following Ex4 treatment (Fig 4C).

### Histological analysis

**Fig 5:**
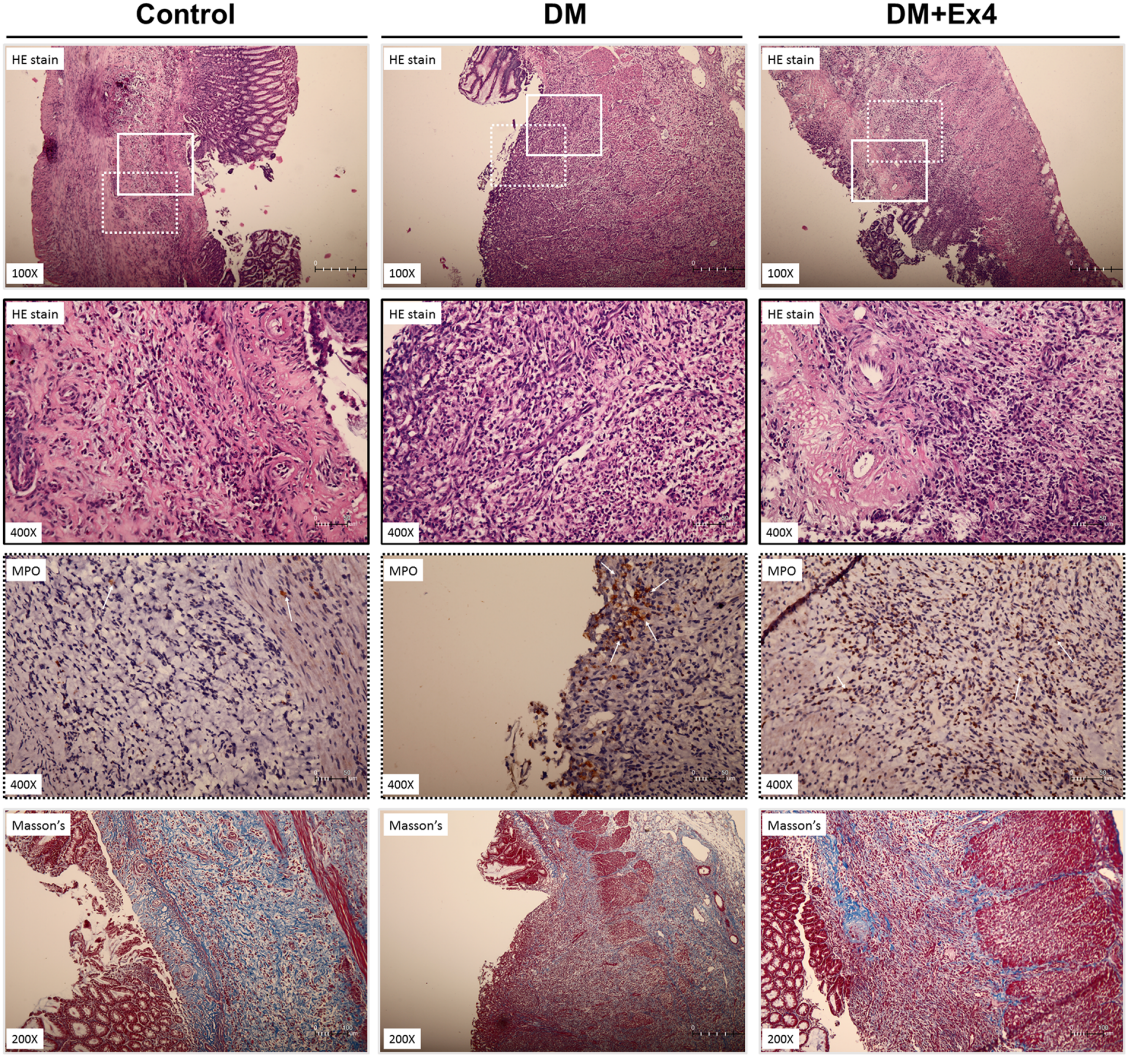
Representative histological sections of gastric ulcers in the three treatment groups at day 7 after creation of gastric ulcer. The upper two panels are tissue stained with hematoxylin & eosin (HE) stain, the third panel is immunohistochemical staining for myeloperoxidase, and the forth panel is tissue stained with Masson’s trichrome stain. Solid-line boxes indicate the magnified view (400x) of HE stained tisses, in which more polymorphonuclear leukocyte infiltration. Dotted-line boxes highlight the immunostaining of myeloperoxidase (arrows) in the peri-ulcer gastric tissue. The Masson’ trichrome stain shows fragmented and disorganized collagen fibers in the peri-ulcer gastric tissue of diabetic rats. Histological sections were performed in 6 different animals in each group.

At day 7 after creation of gastric ulcer, the hematoxylin & eosin (HE) and Masson’s trichrome stains showed the formation granulation tissue and capillaries in the gastric wall of control rats (Fig 5). In diabetic rats, infiltration of polymorphonuclear leukocytes (PMN) was markedly increased and the expression of myeloperoxidase was enhanced in the ulcer area (Fig 5). Masson’s trichrome stain indicated fragmented and disorganized collagen fibers in the ulcer bed of diabetic animals, suggesting the delayed formation of granulation (Fig 5). Systemic administration of Ex4 not only suppressed the cellular infiltration in the peri-ulcer tissue, but also attenuated the tissue levels of myeloperoxidase in diabetic animals (Fig 5).

## Discussion

Defects on the gastrointestinal mucosa and submucosal layers trigger the inflammatory reactions by releasing pro-inflammatory cytokines such as TNF-α and IL-1β, thereby activating the regional epithelial cells, endothelial cells and fibroblasts [18]. Healing of peptic ulcer involves complex tissue remodeling processes, including cell migration, proliferation, re-epithelialization, angiogenesis, and matrix deposition, leading to scar formation [19]. The re-epithelialization and development of granulation tissue at the ulcer base are modulated by the release and interactions of growth factors (i.e. epidermal growth factors, hepatocyte growth factor, transforming growth factorsα/β and vascular endothelial growth factor). Therefore, the tight regulation between inflammation and cell migration/proliferation process surrounding the ulcer region determines the quality and speed of ulcer healing. Diabetes has been recognized as one of the most important clinical risk factors responsible for the impaired wound healing due to generation of excessive reactive oxygen species, inhibition of endothelial cell/myofibroblast migration and proliferation, attenuation of angiogenic response and enhancement of pro-inflammatory reaction [20]. Experimental studies showed that gastric mucosal microcirculation was reduced and the expressions of vascular endothelial markers were suppressed at the ulcer borders in diabetic animals [6,21], suggesting that regional angiogenesis and neovascularization are impaired during ulcer wound healing in chronic hyperglycemia. In addition, inflammatory reaction in the gastrointestinal walls is enhanced in diabetic patients, resulting in high prevalence (up to 47%) of silence active gastric ulcers or erosion in these patients [22]. Animal studies showed that prolonged healing of peptic ulcer in diabetic rats was associated with increased gastric mucosal expression and release of TNF-α and IL-1β [6], evidenced that amplification of local inflammation is also responsible for delayed healing process of PUD in diabetes.

Injection of acetic acid into the subserosal layer of gastric wall generates a single chronic ulcer lesion and may remain unhealed for more than 250 days in rat stomach [23]. The ulcers penetrate the entire gastric wall and induce pathological features that are highly resembled PUD in human subjects [14]. Consistent with the previous reports [6,24], the ulcer size was significantly larger in the diabetic rats at 7 days after focal injection of acetic acid in this study. Our results showed that creation of chronic peptic ulcer reduced the pH value of gastric juice and induced physiological stress by stunting the body weight gain (S1 Table). Creation of chronic gastric ulcer also induced the tissue expressions of CD11b and HO-1, indicating the ongoing process of leukocyte infiltration and regional inflammatory response (S3 Fig). The impaired healing of gastric ulcer in diabetic rats was associated with suppressed tissue expression endothelial cell markers (i.e. eNOS and peNOS), enhanced pro-inflammatory reactions (i.e. IL-1β, IL-6 and myeloperoxidase activity, S2 and S4 Figs) and reduced regenerative activity (i.e. MMP-2, IL-10 and cAMP, S3 and S4 Figs). The increased expression of eNOS in the gastric tissue indicates a normal healing process of peptic ulcer [25] and enhancement of eNOS activity may further accelerate healing of gastric ulcers [26]. Supplement of eNOS substrate, L-arginine significantly decreased cell apoptosis in the injured gastric mucosa and improved mucosal regeneration in wild-type rats [27]. Systemic and regional inflammatory reaction is another key factor for peptic ulcer healing. Infiltration of macrophages and neutrophils, and the release of pro-inflammatory cytokines (such as TNF-α, IL-1β and MCP-1) significantly affect the healing and recurrence of peptic ulcer [28]. Experimental studies also showed that the prolongation of the ulcer healing in diabetic animals was associated with an increase in gastric mucosal expression and release of TNF-α, IL-1β and reactive oxygen species [6,29]. The acute inflammatory phase of ulcer healing is followed by proliferative phase, in which gelatinases (MMP-2 and MMP-9) are activated to degrade collagens and gelatin for tissue regeneration [17]. MMP-2 became prominent in the ulcer margin from 2 to 21 days after injection of acetic acid in rats, suggesting MMP-2 is an appreciable marker in gastric ulceration and healing [30]. Our study showed that the expressions of eNOS and its phosphorylated form were significantly suppressed in the regenerated tissue surrounding gastric ulcer of diabetic rats (S3 Fig), which is supported by the general concept that chronic hyperglycemia impairs endothelium-derived NO biosynthesis by increased expression of caveolin-1, overproduction of reactive oxygen species (ROS), and eNOS uncoupling [31]. In this study, we also found that the gastric tissue surrounding the chronic ulcer in diabetic rats expressed higher levels of IL-1β, IL-6 and superoxide anions (S2 and S4 Figs). The myeloperoxidase assay further confirmed that the enzymatic activity of activated mononuclear cells was enhanced in the gastric wall of rats with chronic hyperglycemia. These findings are consistent with the evidence that diabetes mellitus enhances generation of oxidative stress (superoxide anions and and oxidative-free radicals) [32] and pro-inflammatory response due to infiltration and activation of inflammatory cells [33]. The suppressed expression and tissue concentration of IL-10 further indicated that the anti-inflammatory reactions were inversely attenuated in diabetic animals [16]. The summation of impaired pro-angiogenesis and amplification of inflammatory responses in the gastric ulcer of diabetic rats thus led to the delayed healing process of chronic ulcer evidenced by MMP-2 expression and ulcer area (S1 Fig).

Experimental studies indicated that supplement of Glp-1 analogues or dipeptidyl peptidase-4 (DPP4) inhibitors restores mucosal damage in the gastrointestinal tract [34,35]. Clinical observational study also found that the expression of Glp-1R was significantly reduced in the gastric mucosa of patients with type 2 diabetes [36], which might contribute to impaired mucosal proliferation. Therefore, the investigation of the regenerative effect of Glp-1 analogues (such as exendin-4) on the healing of gastric ulcer in diabetic subjects is clinically sound. Exendin-4 has been recognized to mediate pro-angionenic and anti-inflammatory responses independent from its hypoglycemic effect that may accelerate wound healing [13,37]. Our study group has recently reported that parenteral administration of Ex4 in diabetic rats significantly enhanced the closure of excisional wound by promoting angiogenesis (expressions of VEGFR-2, p-eNOS, HIF-1 and mobilization of endothelial progenitor cells) and suppression of inflammatory reactions (IL-6 and superoxide anions) in the wound tissues [11]. Extended logically from these recent reports, the regenerative effect of Ex4 on wound repair was therefore tested in the healing of chronic gastric ulcer in diabetes. The results of our study demonstrated that treatment with Ex4 significantly restored the protein expression of eNOS/p-eNOS, attenuated the inflammatory reaction and generation of reactive oxygen species in the peri-ulcer region of stomach harvested from diabetic rats (S3 and S4 Figs). In line with the well-characterized proangiogenic and anti-inflammatory properties of Ex4, our study suggests that these pleiotropic protective effects may be the key biological mechanisms for enhanced healing of gastric ulcer in diabetes. Histological examination of gastric ulcer also supports that the pro-inflammatory response was suppressed in the diabetic rats treated with Ex4. The improvement in the regeneration process results in the activation of MMP-2 and formation of granulation tissue in ulcer base, thereby leading to reduced ulcer size.

There are several limitations of this study that need to be addressed. First, Glp-1 analogues are mainly applied to patients with type II diabetes, but streptozotocin-induced (type I) diabetic rat model was used in this study. As the main objective of our study was to examine the pleiotropic effect of Glp-1R agonism on the healing of gastric ulcer in subjects with hyperglycemia, streptozotocin-induced experimental model was conveniently chosen to represent the physiological conditions of chronic hyperglycemia. Second, administration of Ex4 did not result in hypoglycemic effect in diabetic rats. We speculated that the lack of hypoglycemic effect of Ex4 in this experimental model might due to suboptimal dose of Ex4 was used [11], and clinical studies also indicate the less significant clinical glycemic controlling effect in type I diabetic patients [38]. Nevertheless, the absence of hypoglycemic effect further confirms the independent pleiotropic effect of Ex4 in the healing of gastric ulcer. Due to the concern of development of hypoglycemia, this study did not test the proliferative effects of Ex4 on the healing of gastric ulcer in non-diabetic animals. Third, acetic acid-induced chronic ulcer might not be perfectly representative of acute acid- or NSAID-related PUD. The application of Glp-1R analogues in acute ulcers may need further investigation in other experimental models. Forth, the regenerative effects of Ex4 on chronic ulcer healing were not compared with other standard therapies, such as antacid drugs. Since Glp-1R agonists or analogues have not been tested in diabetic subjects with gastric or duodenal ulcers, the results of this study may provide fundamental evidence for the subsequent comparative studies between different antipeptic ulcer therapies in diabetes. Finally, this was a proof-of-concept experimental study, further investigation into more detailed molecular mechanisms is warranted.

In conclusion, impaired healing of chronic gastric ulcer in subjects with diabetes is potentiated by parenteral administration of a Glp-1R analogue Ex4. The pleiotropic regenerative effect of Ex4 on peptic ulcer healing is independent from the hypoglycemic effect or direct neutralization of gastric acid, but is most likely mediated by promoting pro-angiogenic reaction and suppressing regional inflammation and oxidative stress in the peri-ulcer tissue, thereby enhancing tissue granulation and closure of ulcerative wounds. These results would support the clinical application of Glp-1R analogues as supplementary hypoglycemic agents in the antipeptic ulcer medication.

## Supporting information

S1 Fig Representative photographies and quantitative mesurement of gastric ulcer in controls and diabetic rats. (PDF)

S2 Fig Quantification of tissue concentrations of inflammatory cytokines in the peri-ulcer gastric tissues at day 7 after creation of gastric ulcer in controls, DM and DM+Ex4 rats. (PDF)

S3 Fig Expressions of inflammation-related and angiogenesis/remodeling-related proteins in the peri-ulcer gastric tissues obtained from the three treatment groups at day 7 after creation of gastric ulcer. (PDF)

S4 Fig Measurement of myeloperoxidase activity in the peri-ulcer tissues of the three treatment groups at day 7 after creation of gastric ulcer. (PDF)

S1 Table Physiological parameters and blood tests. (PDF)
