# Supplementary material for: Exendin-4, a glucagon-like peptide-1 analogue accelerates healing of chronic gastric ulcer in diabetic rats
Source: PLoS One. 2017 Nov 2;12(11):e0187434. doi: 10.1371/journal.pone.0187434 (PMC5667749; doi:10.1371/journal.pone.0187434)
Supplement: S1 Fig — (PDF) [file pone.0187434.s001.pdf]

Ulcer base (cm2)

PUD	PUDE	PUDD	PUDDE
0.257	0.043	0.367	0.086
0.079	0.156	0.357	0.338
0.054	0.11	0.343	0.215
0.071	0.371	0.604	0.16
0.064	0.211	0.56	0.1
0.097	0.326	0.968	0.179
	0.192	0.279	
		0.374	

PUD: control; PUDE: control+Ex4; PUDD: DM; PUDDE: DM+Ex4

Ulcer border (cm2)

PUD	PUDE	PUDD	PUDDE
0.914	0.412	1.05	0.364
0.324	0.768	1.003	0.995
0.215	0.285	1.209	1.259
0.359	1.254	1.162	0.674
0.551	0.527	1.35	0.446
0.31	0.866	1.744	0.673
	0.496	0.685	
		1.332	

PUD: control; PUDE: control+Ex4; PUDD: DM; PUDDE: DM+Ex4
