# Supplementary material for: Exendin-4, a glucagon-like peptide-1 analogue accelerates healing of chronic gastric ulcer in diabetic rats
Source: PLoS One. 2017 Nov 2;12(11):e0187434. doi: 10.1371/journal.pone.0187434 (PMC5667749; doi:10.1371/journal.pone.0187434)
Supplement: S2 Fig — (PDF) [file pone.0187434.s002.pdf]

Tissue Il-1B (pg/ml)

PUD	PUDD	PUDDE
103.53	333.48	55.24
115.37	194.04	73.2
97.02	333.48	125.82
120.48	185.81	79.83
149.63	181.83	117.9
166.74	117.9	225.81
117.9		246.25
120.48		

PUD: control; PUDD: DM; PUDDE: DM+Ex4

Tissue IL-6 (pg/ml)

PUD	PUDD	PUDDE
6.7198	6.5465	5.94
6.6331	6.8064	6.1566
6.3299	7.1099	5.7234
6.2433	6.9363	7.0553
6.5032	7.6728	6.5465
6.1566	6.3299	5.94
6.7198		6.3299
		7.0449
		4.9003

PUD: control; PUDD: DM; PUDDE: DM+Ex4

Tissue IL-10 (pg/ml)

PUD	PUDD	PUDDE
683.56	203.51	451.67
732.61	371.25	397.24
563.55	172.48	555.74
332.72	93.56	332.47
711.43	283.31	310.93
392.27	273.75	614.82
1140	310.66	703.59
579.32		359.34
210.83		263.49

PUD: control; PUDD: DM; PUDDE: DM+Ex4

Tissue MCP-1 (pg/ml)

PUD	PUDD	PUDDE
119.76	119.76	97.62
303.95	119.76	206.3
119.76	151.98	95.1
119.76	303.95	106.3
		119.76
		119.76
		119.76

PUD: control; PUDD: DM; PUDDE: DM+Ex4
