# Supplementary material for: Exendin-4, a glucagon-like peptide-1 analogue accelerates healing of chronic gastric ulcer in diabetic rats
Source: PLoS One. 2017 Nov 2;12(11):e0187434. doi: 10.1371/journal.pone.0187434 (PMC5667749; doi:10.1371/journal.pone.0187434)
Supplement: S3 Fig — (PDF) [file pone.0187434.s003.pdf]

Western blot densitometry (ImageJ)

	peNOS	eNOS	HO-1	CD11b	IL10	Caspase3	MMP2
Sham	1.21	2.14	2.89	1.08	1.91		0.83
Sham	1.00	1.00	1.00	1.00	1.00	1.00	1.00
Sham	1.00	1.00	1.00	1.00	1.00	1.00	1.00
PUD				3.74	2.74	1.47	1.39
PUD	6.46	5.82	4.10	7.90	2.34	0.85	1.84
PUD	5.13	5.52	13.11	4.52	2.90	1.53	1.22
PUD			6.47	4.19	2.13	0.96	1.58
PUD	2.65	5.07	16.96	7.61	2.06	1.15	1.24
PUD	1.94	3.39	12.68	4.57	2.78	1.50	1.36
PUDD	2.06	2.06	9.77	3.53	2.13	1.53	1.41
PUDD	2.16	3.74	10.19	6.69	1.57	0.84	1.20
PUDD	0.12	3.32	27.75	3.74	1.56	1.55	0.91
PUDD	1.31	2.39	15.04	3.91	1.24	1.27	1.01
PUDD	1.21	2.61	31.67	8.28	1.36	0.70	0.52
PUDDE	4.49	3.73	8.14	3.53	3.56	0.77	1.62
PUDDE	3.85	4.16	9.36	6.47	3.28	1.20	1.88
PUDDE	5.20	2.99	3.91	3.18	2.13	0.81	1.52
PUDDE	4.72	2.73	5.76	3.99	3.54	0.69	1.92
PUDDE	1.97	3.49	10.94	2.97	1.84	1.14	1.37
PUDDE	3.15	3.94	8.32	5.58	1.68	0.60	1.27

PUD: control; PUDE: control+Ex4; PUDD: DM; PUDDE: DM+Ex4
