# Supplementary material for: Exendin-4, a glucagon-like peptide-1 analogue accelerates healing of chronic gastric ulcer in diabetic rats
Source: PLoS One. 2017 Nov 2;12(11):e0187434. doi: 10.1371/journal.pone.0187434 (PMC5667749; doi:10.1371/journal.pone.0187434)
Supplement: S4 Fig — (PDF) [file pone.0187434.s004.pdf]

Tissue cAMP (pmol/mg protein)

Sham	PUD	PUDE	PUDD	PUDDE
13.352	9.034	8.01	10.473	11.966
9.486	7.394	7.146	10.682	10.552
9.334	6.412	5.358	9.1108	10
10.184	8.618	7.326	8.9238	9.378
17.44	13.414	6.742	9.823	10.306
	8.14	11.214	14.479	10.812
	8.79	8.626	17.732	13.56
	9.694	5.436	9.2549	5.546
	9.45	3.434	13.069	8.388
	15.972	9.868		6.186
	11.196	8.002		
	7.01	9.868		
	6.458	8.002		

PUD: control; PUDE: control+Ex4; PUDD: DM; PUDDE: DM+Ex4

Myeloperoxidase activity (U/g protein)

Sham	PUD	PUDE	PUDD	PUDDE
1.402	2.6214	2.3632	3.0252	2.0984
1.688	2.8846	2.1514	3.2452	2.13
1.642	2.8954	1.2919	3.0149	2.3798
	1.8243	0.9122	3.24	2.4272
	1.4385	0.8282	2.5478	2.3427
		1.8443	3.2143	2.0287
		2.3547		

PUD: control; PUDE: control+Ex4; PUDD: DM; PUDDE: DM+Ex4

Tissue superoxide anions (µmol/min/g protein)

Sham	PUD	PUDE	PUDD	PUDDE
85	196	154	290	270
112	235	221	297	170
93	242	170	217	165
137	280	160	311	193
		211	201	200
		264	392	212
		254	343	169
			282	

PUD: control; PUDE: control+Ex4; PUDD: DM; PUDDE: DM+Ex4
