# Supplementary material for: Exendin-4, a glucagon-like peptide-1 analogue accelerates healing of chronic gastric ulcer in diabetic rats
Source: PLoS One. 2017 Nov 2;12(11):e0187434. doi: 10.1371/journal.pone.0187434 (PMC5667749; doi:10.1371/journal.pone.0187434)
Supplement: S1 Table — (PDF) [file pone.0187434.s005.pdf]

Gastric juice pH

PUD	PUDE	PUDD	PUDDE
6	3	4	3
2	3	3	3
2	4	3	3
3	4	3	3
3	3	2	3
4	3	2	3
3	3	2	2
3	3	5	2
	3	3	4
	3	3	3
	4		

PUD: control; PUDE: control+Ex4; PUDD: DM; PUDDE: DM+Ex4

Body weight gained (gm)

PUD	PUDE	PUDD	PUDDE
30	0	-25	-25
35	10	-10	-15
10	5	-30	-30
25	5	-5	-30
-10	10	-10	-20
-20	35	-25	-20
15	10	-20	-10
5	5	-20	-40
10	20	-20	-50
-40		-15	-35
-35			
0			

PUD: control; PUDE: control+Ex4; PUDD: DM; PUDDE: DM+Ex4

Blood glucose (mg/dl)

Sham	PUD	PUDE	PUDD	PUDDE
144	178	108	198	469
148	145	126	194	222
148	143	118	427	381
225	156	112	363	445
	178	136	510	489
	173	147	431	216
	149	116	184	348
	106	119		478
		102		
		123		
		130		

PUD: control; PUDE: control+Ex4; PUDD: DM; PUDDE: DM+Ex4

Serum IL-1B

Sham	PUD	PUDD	PUDDE
903.6	828.7	696.8	379.9
865.3	759.9	903.6	471.8
611.9	561.1	1224.0	305.9
727.7	514.5	1029.0	1159.0
	667.3	2149.0	696.8
	471.8	561.1	379.9
	828.7		561.1
			1147.0
			134.3

PUD: control; PUDE: control+Ex4; PUDD: DM; PUDDE: DM+Ex4

Sham	PUD	PUDD	PUDDE
119.8	119.8	119.8	97.6
152.0	304.0	119.8	119.8
152.0	119.8	152.0	119.8
152.0	119.8	304.0	119.8
